# The Association between Leisure-Time Physical Activity and Risk of Undetected Prediabetes

**DOI:** 10.1155/2017/4845108

**Published:** 2017-03-06

**Authors:** Jia Wang, Yili Wu, Feng Ning, Chaoying Zhang, Dongfeng Zhang

**Affiliations:** ^1^Department of Epidemiology and Health Statistics, The Medical College of Qingdao University, No. 38 Dengzhou Road, Qingdao 266021, China; ^2^Department of Chronic Noncommunicable Diseases, Qingdao Centers for Disease Control and Prevention, Qingdao 266033, China

## Abstract

*Aims*. The purpose of the study was to assess the effects of leisure-time physical activity on undetected prediabetes.* Methods*. Data from the National Health and Nutrition Examination Survey 2007–2012 were used in our analyses. Logistic regression was conducted to estimate the odds ratios (ORs) with 95% confidence intervals (CIs) of prediabetes associated with leisure-time physical activity.* Results*. A total of 8204 subjects were eligible for our analyses. For all subjects, high level of total leisure-time physical activity (OR = 0.78, 95% CI: 0.66, 0.94) and low level of vigorous leisure-time physical activity (OR = 0.72, 95% CI: 0.58, 0.90) were inversely associated with the risk of prediabetes in multivariate-adjusted model. For subjects under 45 years of age, high level of total leisure-time physical activity (OR = 0.78, 95% CI: 0.61, 0.99) and low (OR = 0.61, 95% CI: 0.45, 0.83) and high (OR = 0.72, 95% CI: 0.53, 1.00) level of vigorous leisure-time physical activity were associated with a decreased risk of prediabetes. In the 45 to 65 age group, only high level of total leisure-time physical activity (OR = 0.73, 95% CI: 0.57, 0.95) had protective effect on prediabetes.* Conclusions*. Leisure-time physical activity may be associated with a decreased risk of prediabetes.

## 1. Introduction

Prediabetes refers to the condition in which blood glucose concentration is higher than normal but not high enough for a diagnosis of diabetes and indicates an increased risk for the future development of diabetes and associated complications [1–3]. In 2012, the estimated percentage and number of US adults with prediabetes were 37% and 86 million, respectively [4]. Results from the Health Survey for England revealed that the prevalence of prediabetes increased from 11.6% to 35.3% during 2003–2011 in England [5]. A cross-sectional survey in a nationally representative sample of Chinese adults showed that the incidence of prediabetes was 50.1%, which represented up to 493.4 million patients with prediabetes in China in 2010 [6]. Even with a high prevalence, the awareness rate of prediabetes was very low. Research from the National Health and Nutrition Examination Survey (NHANES) showed that the estimated awareness rate of prediabetes was less than 14% in the US during 2005–2010 [7]. Hence, it is very important to pay attention to and prevent prediabetes considering the significant public health impact.

Physical activity is beneficial to the prevention of many chronic diseases [8]. A meta-analysis based on prospective studies found that various types of physical activity were beneficial to the prevention of diabetes, and the risk of diabetes decreased by 15% for 20 MET-hours/week increment of leisure-time physical activity [9]. Furthermore, several randomized controlled trials reported that physical activity could improve insulin sensitivity and glucose tolerance and then delay the onset of diabetes in subjects with prediabetes [10–13]. Leisure-time physical activity (sports, exercise and recreational activity, etc.) has more advantages than work-related physical activity. It allows a more flexible schedule than work-related physical activity and cannot cause strain associated with vigorous-intensity work. Therefore, leisure-time physical activity may be a good choice for the prevention of chronic diseases. But there are few studies about the association between leisure-time physical activity and the risk of prediabetes. Thus, we performed analyses on a subsample of NHANES 2007–2012 to assess the association between leisure-time physical activity and the risk of prediabetes.

## 2. Methods

### 2.1. Study Population

Data from NHANES 2007–2012 were used in this study. With a complex, multistage probability design, NHANES examines a nationally representative sample of the US civilian noninstitutionalized population of all ages. NHANES participants undergo an at-home health interview and a clinic examination in a specially designed and equipped mobile examination center (MEC). The clinical examination lasts 3-4 hours and all data collection methods are standardized to minimize site-specific bias. All participants provided informed consent for both the at-home interview and MEC examination [14]. The response rates of interviewed sample were 78.4%, 79.4%, and 72.6% for 2007-2008, 2009-2010, and 2011-2012, respectively. And the response rates of examined sample were 75.4%, 77.3%, and 69.5% for 2007-2008, 2009-2010, and 2011-2012, respectively [15]. A total of 30,442 individuals participated in the NHANES during 2007–2012. We excluded individuals with hemoglobin A1c (HbA1c) ≥ 6.5% (≥48 mmol/mol) (1376) or self-reported diabetes or receiving diabetes treatment (1241) and individuals with self-reported prediabetes (419) or self-reported borderline diabetes (164). We further excluded the participants with a history of congestive heart failure, coronary heart disease, angina pectoris, heart attack, emphysema, and chronic bronchitis. Besides, participants without data about body mass index (BMI), race, educational level, smoking status, alcohol consumption, dietary pattern, daily total energy intake, physical activity, and measurements of blood pressure (BP) and HbA1c were also excluded. Finally, 8204 subjects between 20 and 65 years old were included in our analyses.

### 2.2. Prediabetes Assessment

Prediabetes was defined according to HbA1c criteria of American Diabetes Association (ADA) [1]. In 2015, ADA recommended that the A1c test should be performed using a method that was certified by the National Glycohemoglobin Standardization Program (NGSP) and standardized or traceable to the Diabetes Control and Complications Trail (DCCT) reference assay [1]. By reviewing the NHANES laboratory and participant HbA1c data, the NGSP group concluded that the NHANES laboratories met the NGSP criteria for bias and precision [16]. And participants with an HbA1c level of 5.7–6.4% (39–46 mmol/mol) were classified as having prediabetes [1].

### 2.3. Physical Activity Assessment

Each NHANES participant completed a physical activity questionnaire which was based on the Global Physical Activity Questionnaire (GPAQ) [17]. The physical activity questionnaire included questions related to daily activities and leisure-time activities. The activity information was collected before the physical examination in the home using the Computer-Assisted Personal Interviewing (CAPI) (interviewer administered) system [17]. Vigorous-intensity activities are activities that require hard physical effort and cause large increases in breathing or heart rate, and moderate-intensity activities are activities that require moderate physical effort and cause small increases in breathing or heart rate [17]. Work-related physical activity refers to paid or unpaid work, studying or training, household chores, and yard work. Leisure-time physical activity refers to sports, fitness, and recreational activities. The suggested metabolic equivalent (MET) scores for vigorous work-related physical activity, moderate work-related physical activity, walking or bicycling for transportation, vigorous leisure-time physical activity, and moderate leisure-time physical activity were 8.0, 4.0, 4.0, 8.0, and 4.0, respectively [17]. The average number of hours per week spent in each activity was multiplied by the suggested MET scores to get an estimate of MET-hours per week. MET-hours per week of vigorous and moderate work-related physical activity were summed to obtain an estimate of total work-related physical activity. MET-hours per week of vigorous and moderate leisure-time physical activity were summed to obtain an estimate of total leisure-time physical activity.

### 2.4. Covariates

Demographic information included age, gender, race (Mexican American, other Hispanic, Non-Hispanic White, Non-Hispanic Black, and other race), and educational level (less than 9th grade, 9th–11th grade, high school graduate/GED or equivalent, some college or AA degree, and college graduate or above). Other covariates included BMI, BP, smoking status (smoking at least 100 cigarettes in life or not), alcohol consumption (having at least 12 alcohol drinks/year or not), dietary pattern (excellent healthy, very good healthy, good healthy, fair healthy, and poor healthy), and daily total energy intake. Educational level was divided into <high school, high school, and >high school. Weight (kg) and height (cm) were measured according to standard procedures and BMI was calculated as weight in kilograms divided by height in meters squared [18]. The BMI was divided into <25.0, 25.0 to <30.0 and ≥30.0 kg/m^2^. After resting quietly in a seated position for 5 minutes and determining the maximum inflation level, three consecutive BP readings were obtained [19]. The average value of three measurements was calculated to identify hypertension. Participants were identified as hypertensive if the mean systolic BP was ≥140 mmHg or diastolic BP was ≥90 mmHg or receiving antihypertensive medicine [20]. All NHANES participants were eligible for two 24-hour dietary recall interviews. The first dietary recall interview was collected in person in the MEC and the second interview was collected by telephone 3 to 10 days later [21]. Daily total energy intake was calculated as the mean of the total energy intake in the first and second interview.

### 2.5. Statistical Analysis

*t*-tests were used to compare the mean levels of continuous variables between subjects with and without prediabetes if the variables conformed to the normal distribution. Nonparametric tests were used to compare the averages of continuous variables between subjects with and without prediabetes if the variables were nonnormal data. Chi-square tests were used to compare the prevalences of categorical variables between subjects with and without prediabetes. The MET-hours per week of each leisure-time physical activity were divided into tertiles based on the distribution in the study sample. The tertiles of each leisure-time physical activity represented low, moderate, and high level of leisure-time physical activity, respectively, with 0 MET-hours per week as the referent group. Logistic regression analyses were conducted to assess the associations between total, vigorous, and moderate leisure-time physical activity and the risk of prediabetes, respectively. Crude odds ratios (ORs), age- and gender-adjusted ORs, and multivariate-adjusted ORs (age, gender, BMI, race, educational level, smoking status, alcohol consumption, dietary pattern, daily total energy intake, hypertension, total work-related physical activity, and walking or bicycling for transportation) were calculated from logistic regression analyses with 95% confidence intervals (CIs). Then, the above-mentioned logistic regression analyses were conducted separately by age (<45 and ≥45) to assess the protective effects of total, vigorous, and moderate leisure-time physical activity on prediabetes in different age groups.

All statistical analyses were performed with STATA version 12.0 (Stata Corporation, College Station, TX, USA). All reported probabilities (*P* values) were two-sided with *P* < 0.05 considered statistically significant.

## 3. Results

Characteristics of subjects by prediabetes status were shown in Table 1. Of the 8204 subjects without diabetes and self-reported prediabetes, 1914 (23.33%) were identified as having undetected prediabetes based on HbA1c criteria. The percentage of undetected prediabetes was significantly higher in the 45- to 65-year group. Subjects with prediabetes tended to be male and non-Hispanic Black. Subjects with prediabetes were significantly more likely to be obese and hypertensive than controls. Compared with normal subjects, those with prediabetes received a lower level of education. The proportion of subjects who smoked at least 100 cigarettes in life was higher in the group with prediabetes. The proportion of subjects who had at least 12 alcohol drinks per year was lower in the group with prediabetes. The proportion of subjects who had excellent and very good healthy dietary pattern was significantly lower in the group with prediabetes. The daily total energy intake in subjects with prediabetes was significantly lower than that in controls. Total, vigorous, and moderate leisure-time physical activity were significantly lower in subjects with prediabetes. However, no statistically significant differences were found in terms of total work-related physical activity and walking or bicycling for transportation between subjects with and without prediabetes.

The ORs (95% CIs) of prediabetes according to category of leisure-time physical activity for all subjects were shown in Table 2. The crude ORs of prediabetes indicated that any level of total, vigorous, and moderate leisure-time physical activity was associated with a decreased risk of prediabetes (OR = 0.46–0.77). After adjustment for age and gender, the results were consistent with the crude ORs (OR = 0.61–0.80). Additional adjustment for BMI, race, educational level, smoking status, alcohol consumption, dietary pattern, daily total energy intake, hypertension, total work-related physical activity, and walking or bicycling for transportation, high level of total leisure-time physical activity (OR = 0.78, 95% CI: 0.66, 0.94), and low level of vigorous leisure-time physical activity (OR = 0.72, 95% CI: 0.58, 0.90) were inversely associated with the risk of prediabetes, respectively. However, the protective effect of moderate leisure-time physical activity on prediabetes was no longer statistically significant after adjustment for more covariates.

The associations between leisure-time physical activity and the risk of prediabetes in different age groups were shown in Table 3. For subjects younger than 45 years of age, any level of total and vigorous leisure-time physical activity was associated with a decreased risk of prediabetes in unadjusted model and age- and gender-adjusted model. Compared with no moderate leisure-time physical activity, the low level of moderate leisure-time physical activity was inversely associated with the risk of prediabetes in unadjusted model and age- and gender-adjusted model. Additional adjustment for BMI, race, educational level, smoking status, alcohol consumption, dietary pattern, daily total energy intake, hypertension, total work-related physical activity, and walking or bicycling for transportation and high level of total leisure-time physical activity were inversely associated with the risk of prediabetes (OR = 0.78, 95% CI: 0.61, 0.99). In multivariate-adjusted model, the ORs (95% CIs) of prediabetes were 0.61 (0.45, 0.83), 0.76 (0.56, 1.03), and 0.72 (0.53, 1.00) for the lowest through the highest tertile of vigorous leisure-time physical activity. However, the inverse association between low level of moderate leisure-time physical activity and the risk of prediabetes was not statistically significant in multivariate-adjusted model.

In the 45- to 65-year group, any level of total, vigorous, and moderate leisure-time physical activity was associated with a decreased risk of prediabetes in unadjusted model and age- and gender-adjusted model. Additional adjustment for BMI, race, educational level, smoking status, alcohol consumption, dietary pattern, daily total energy intake, hypertension, total work-related physical activity, and walking or bicycling for transportation, however, largely attenuated the protective effects of vigorous and moderate leisure-time physical activity on prediabetes. The inverse association between high level of total leisure-time physical activity and prediabetes was slightly attenuated but remained significant in the multivariate-adjusted model (OR = 0.73, 95% CI: 0.57, 0.95). But the inverse associations of low and moderate level of total leisure-time physical activity with prediabetes were not statistically significant in multivariate-adjusted model.

## 4. Discussion

Our study used a large national database to explore the associations between leisure-time physical activity and the risk of undetected prediabetes. Our analyses included 8204 nonpregnant adults without diabetes and self-reported prediabetes. Among those seemingly healthy subjects, 1914 (23.33%) were identified as prediabetics. For all subjects, total, vigorous, and moderate leisure-time physical activity were associated with a decreased risk of prediabetes in unadjusted model and age- and gender-adjusted model. What is more, the protective effects of high level of total leisure-time physical activity and low level of vigorous leisure-time physical activity on prediabetes were still statistically significant in multivariate-adjusted model. To further assess the effects of leisure-time physical activity on prediabetes in different age groups, we conducted logistic regression in under 45-year and 45- to 65-year group, respectively. And we found that the protective effect of vigorous leisure-time physical activity on prediabetes was more pronounced in under 45-year group.

As our research, some other studies had suggested that physical activity might reduce the risk of prediabetes. A population-based survey conducted in a predominantly rural Demographic Surveillance Site in eastern Uganda reported that person who met the WHO minimum recommended physical activity level had a significantly lower likelihood of abnormal glucose regulation [22]. Another study based on the NHANES 2003–2006 assessed the relationship between objectively measured physical activity and prediabetes [23]. The results indicated that, compared with subjects within the lowest tertile, those within the highest tertile were 0.77 times as likely to have prediabetes when controlling for BMI alone [23]. However, the inverse association became nonstatistically significant after controlling for more covariates [23]. But very few studies have looked at the association between specific type of physical activity and the risk of prediabetes. Compared to work-related physical activity, leisure-time physical activity has more advantages, such as being more flexible, entertaining, and relaxing. Therefore, we speculated that leisure-time physical activity may play a key role in the prevention of prediabetes. It is necessary to conduct this research to explore the association between leisure-time physical activity and the risk of prediabetes.

There are several mechanisms underlying the association between physical activity and the risk of prediabetes. First, physical activity can improve energy balance and prevent obesity [24], which is a major risk factor independently related to prediabetes [22, 25]. Second, physical activity can reduce blood glucose and increase insulin sensitivity in people with and without diabetes directly [26–28]. And vigorous physical activity has greater effects on the improvements in insulin sensitivity and reductions in blood glucose than nonvigorous physical activity [28]. This supports our result that the protective effect of vigorous leisure-time physical activity on prediabetes is more pronounced than moderate leisure-time physical activity. Third, glucose transporter 4 (GLUT4) is the predominant glucose transporter isoform expressed in skeletal muscle. The process of glucose uptake into the contracting skeletal muscles is regulated by the translocation of GLUT4 to the plasma membrance and transverse tubules [29]. Long-term physical activity can increase the expression of GLUT4 and the concentration of mitochondrial enzyme and transform fiber type in skeletal muscle, thus reducing the risk of glucose abnormality [30, 31].

People with prediabetes are at high risk, not only to develop diabetes, but also to suffer from adverse cardiovascular (CV) events (myocardial infarction, stroke, and CV death) later in life [2]. Therefore, from clinical perspective, we suggested that people should take more leisure-time physical activity to prevent the occurrence of prediabetes. We hope that increased leisure-time physical activity can effectively increase participants' quality of life.

There are several strengths in our study. The main strength is the large and representative sample included, increasing the statistical power of the study to detect the decrease in risk of prediabetes. Second, we excluded subjects with self-reported prediabetes, who might actively participate in leisure-time physical activity based on the recommendations of healthcare workers. This made the observed associations between leisure-time physical activity and the risk of prediabetes more actual and credible. Third, the protective effects of total leisure-time physical activity and vigorous leisure-time physical activity on prediabetes were still statistically significant after adjustment for total work-related physical activity and walking or bicycling for transportation, increasing the authenticity of the observed associations. Fourth, the anthropometric measurements were collected by trained health technicians through standard procedures, further increasing the accuracy of the results.

However, some limitations in our study have to be mentioned. First, the data we used in our analyses was from a cross-sectional study, which limited the establishment of the causal association between leisure-time physical activity and the risk of prediabetes. Second, we only adopted the HbA1c level as the diagnostic criteria for prediabetes on account of the large number of missing values about fasting plasma glucose (FPG) and oral glucose tolerance test (OGTT) data. We might miss some subjects with prediabetes though the HbA1c test had several advantages over FPG and OGTT, including greater convenience, greater preanalytical stability, and less day-to-day perturbations during stress and illness [1]. Third, the information on physical activity in our analyses was obtained by self-report, which might be subject to recall bias.

## 5. Conclusion

Our study indicates that leisure-time physical activity may decrease the risk of prediabetes. Large cohort studies are needed to verify the potential causal association between leisure-time physical activity and the risk of prediabetes. It is very important to improve the awareness of prediabetes and prevent the onset of prediabetes for the prevention and postponement of diabetes.

## Figures and Tables

**Table 1:** Characteristics of participants by prediabetes status, NHANES 2007–2012, ages 20–65 y.

	Prediabetes	Control	*P* value
Number of subjects (%)	1914 (23.33)	6290 (76.67)	
Gender (%)			0.007
Male	54.02	50.48	
Age (year)	47.64 ± 11.99	38.42 ± 12.57	<0.001
Age group (%)			<0.001
20–44	36.94	67.77	
45–65	63.06	32.23	
BMI (kg/m^2^)	30.58 ± 7.08	27.58 ± 6.06	<0.001
BMI group (%)			<0.001
<25 kg/m^2^	19.85	37.07	
25 to <30 kg/m^2^	34.85	34.44	
≥30 kg/m^2^	45.30	28.49	
Hypertension (%)	18.34	9.33	<0.001
Daily total energy intake (kcal)	2141.34 ± 892.91	2203.72 ± 913.18	0.003
Smoke at least 100 cigarettes in life (%)	46.50	40.67	<0.001
Have at least 12 alcohol drinks/year (%)	74.45	78.08	0.001
Dietary pattern (%)			0.025
Excellent healthy	6.90	8.19	
Very good healthy	18.55	20.49	
Good healthy	42.63	42.37	
Fair healthy	25.97	24.01	
Poor healthy	5.96	4.94	
Educational level (%)			<0.001
<high school	29.05	20.52	
High school	25.76	21.70	
>high school	45.19	57.77	
Race (%)			<0.001
Mexican American	19.70	17.01	
Other Hispanic	11.60	10.68	
Non-Hispanic White	30.51	46.30	
Non-Hispanic Black	30.51	17.11	
Other race	7.68	8.90	
Level of physical activity (MET-hours/week)			
Total leisure-time physical activity	11.66 ± 24.94	16.99 ± 30.04	<0.001
Moderate leisure-time physical activity	5.65 ± 12.72	6.52 ± 13.65	<0.001
Vigorous leisure-time physical activity	6.01 ± 19.34	10.47 ± 24.41	<0.001
Total work-related physical activity	48.20 ± 103.72	47.67 ± 97.48	0.223
Walking or bicycling for transportation	8.01 ± 23.49	7.63 ± 22.82	0.288

Data are means ± SD unless indicated otherwise.

BMI, body mass index; MET, metabolic equivalent.

**Table 2:** Odds ratios of prediabetes according to category of leisure-time physical activity, NHANES 2007–2012, ages 20–65 y.

	Case	Participant	Crude		Model 1		Model 2	
			OR	95% CI	OR	95% CI	OR	95% CI
Total leisure-time physical activity (MET-hours/week)								
None (reference)	1065	3793	1.00		1.00		1.00	
Low (≤12)	357	1700	0.68	0.59, 0.78	0.72	0.63, 0.83	0.88	0.76, 1.03
Moderate (>12 to 28)	258	1249	0.67	0.57, 0.78	0.75	0.64, 0.88	0.96	0.81, 1.14
High (>28)	234	1462	0.49	0.42, 0.57	0.64	0.54, 0.75	0.78	0.66, 0.94
*P*-trend				<0.001		<0.001		0.016
Vigorous leisure-time physical activity (MET-hours/week)								
None (reference)	1564	5882	1.00		1.00		1.00	
Low (≤16)	126	881	0.46	0.38, 0.56	0.61	0.49, 0.75	0.72	0.58, 0.90
Moderate (>16 to 32)	120	733	0.54	0.44, 0.66	0.69	0.55, 0.85	0.81	0.65, 1.02
High (>32)	104	708	0.48	0.38, 0.59	0.67	0.53, 0.84	0.82	0.64, 1.04
*P*-trend				<0.001		<0.001		0.010
Moderate leisure-time physical activity (MET-hours/week)								
None (reference)	1199	4635	1.00		1.00		1.00	
Low (≤6)	220	1209	0.64	0.54, 0.75	0.70	0.59, 0.83	0.91	0.76, 1.08
Moderate (>6 to 14)	255	1230	0.75	0.64, 0.87	0.78	0.66, 0.91	1.02	0.86, 1.21
High (>14)	240	1130	0.77	0.66, 0.90	0.80	0.68, 0.94	0.99	0.83, 1.18
*P*-trend				<0.001		<0.001		0.949

OR, odds ratio; CI, confidence interval; MET, metabolic equivalent.

Model 1 adjusted for age and gender.

Model 2 adjusted for age, gender, race, BMI, educational level, smoking status, alcohol consumption, dietary pattern, daily total energy intake, hypertension, total work-related physical activity, and walking or bicycling for transportation.

**Table 3:** Odds ratios of prediabetes according to category of leisure-time physical activity, stratified by age, NHANES 2007–2012, ages 20–65 y.

	Case	Participant	Crude		Model 1		Model 2	
			OR	95% CI	OR	95% CI	OR	95% CI
	20–44 y							
Total leisure-time physical activity (MET-hours/week)								
None (reference)	352	2062	1.00		1.00		1.00	
Low	130	1037	0.70	0.56, 0.86	0.71	0.57, 0.89	0.88	0.69, 1.11
Moderate	100	789	0.71	0.56, 0.90	0.73	0.57, 0.93	0.90	0.70, 1.17
High	125	1082	0.63	0.51, 0.79	0.66	0.53, 0.83	0.78	0.61, 0.99
*P*-trend				<0.001		<0.001		0.050
Vigorous leisure-time physical activity (MET-hours/week)								
None (reference)	524	3227	1.00		1.00		1.00	
Low	60	660	0.52	0.39, 0.68	0.53	0.40, 0.71	0.61	0.45, 0.83
Moderate	64	529	0.71	0.54, 0.94	0.71	0.54, 0.95	0.76	0.56, 1.03
High	59	554	0.61	0.46, 0.82	0.65	0.49, 0.87	0.72	0.53, 1.00
*P*-trend				<0.001		<0.001		0.007
Moderate leisure-time physical activity (MET-hours/week)								
None (reference)	416	2698	1.00		1.00		1.00	
Low	86	804	0.66	0.51, 0.84	0.66	0.51, 0.85	0.90	0.69, 1.18
Moderate	104	760	0.87	0.69, 1.10	0.91	0.72, 1.16	1.21	0.93, 1.56
High	101	708	0.91	0.72, 1.15	0.92	0.73, 1.17	1.14	0.88, 1.48
*P*-trend				0.195		0.305		0.171

	45–65 y							
Total leisure-time physical activity (MET-hours/week)								
None (reference)	713	1731	1.00		1.00		1.00	
Low	227	663	0.74	0.62, 0.90	0.73	0.61, 0.88	0.88	0.72, 1.07
Moderate	158	460	0.75	0.60, 0.93	0.76	0.61, 0.94	0.98	0.78, 1.23
High	109	380	0.57	0.45, 0.73	0.60	0.47, 0.76	0.73	0.57, 0.95
*P*-trend				<0.001		<0.001		0.047
Vigorous leisure-time physical activity (MET-hours/week)								
None (reference)	1040	2665	1.00		1.00		1.00	
Low	66	221	0.66	0.49, 0.89	0.70	0.52, 0.95	0.86	0.62, 1.18
Moderate	56	204	0.59	0.43, 0.81	0.63	0.46, 0.87	0.79	0.57, 1.12
High	45	154	0.64	0.45, 0.92	0.67	0.47, 0.96	0.86	0.58, 1.26
*P*-trend				<0.001		<0.001		0.135
Moderate leisure-time physical activity (MET-hours/week)								
None (reference)	783	1937	1.00		1.00		1.00	
Low	134	405	0.73	0.58, 0.91	0.74	0.59, 0.93	0.91	0.72, 1.16
Moderate	151	470	0.70	0.56, 0.86	0.69	0.55, 0.85	0.88	0.70, 1.10
High	139	422	0.72	0.58, 0.90	0.71	0.56, 0.89	0.87	0.69, 1.11
*P*-trend				<0.001		<0.001		0.150

OR, odds ratio; CI, confidence interval; MET, metabolic equivalent.

Model 1 adjusted for age and gender.

Model 2 adjusted for age, gender, race, BMI, educational level, smoking status, alcohol consumption, dietary pattern, daily total energy intake, hypertension, total work-related physical activity, and walking or bicycling for transportation.
